# Intratesticular prepubertal‑type mature teratoma in an infant: A case report and mini‑review of the literature

**DOI:** 10.3892/mi.2024.177

**Published:** 2024-07-15

**Authors:** Saman S. Fakhralddin, Rawa M. Ali, Sami Saleem Omar, Rebaz M. Ali, Bryar O. Mohammed, Rawa Amin Karim, Hiwa O. Abdullah, Karukh K. Mohammed, Shvan H. Mohammed, Sasan M. Ahmed, Fahmi H. Kakamad

**Affiliations:** 1 College of Medicine, University of Sulaimani, Sulaymaniyah, Kurdistan 46001, Iraq; 2 Department of Scientific Affairs, Smart Health Tower, Sulaymaniyah, Kurdistan 46001, Iraq; 3 Department of Urology, Sulaymaniyah Surgical Teaching Hospital, Sulaymaniyah, Kurdistan 46001, Iraq; 4 Hospital for Treatment of Victims of Chemical Weapons, Halabja, Kurdistan 46018, Iraq; 5 Rizgary Oncology Center, Erbil, Kurdistan 44001, Iraq; 6 Kscien Organization for Scientific Research, Sulaymaniyah, Kurdistan 46001, Iraq; 7 Department of Oncology, Hiwa Hospital, Sulaymaniyah, Kurdistan 46001, Iraq; 8 Smart Health Tower (Raparin Branch), Rania, Sulaymaniyah, Kurdistan 46001, Iraq; 9 Xzmat Polyclinic, Kalar, Sulaymaniyah, Kurdistan 46001, Iraq

**Keywords:** pediatric, teratoma, testicular tumor, infant

## Abstract

Testicular tumors are rare in children, representing a small percentage of pediatric solid tumors, with an incidence of 2 cases per 100,000 males. Teratomas, which are the most prevalent tumors in infants, may manifest in mature, immature, or malignant forms. While mature teratomas are typically found in the abdomen, intratesticular prepubertal-type teratomas in infants are infrequent. The present study describes the case of an infant with an intratesticular mature teratoma. A 6-month-old male infant presented with right-sided scrotal swelling, which was noted by his parents. There was no family history of similar conditions, and an investigation of his medical history did not reveal any notable findings. A physical examination revealed a non-reducible, solid mass indistinguishable from the right testicle, with no signs of inflammation or systemic symptoms. A scrotal sonography confirmed a large intratesticular cyst. The levels of α-fetoprotein and β-human chorionic gonadotropin were normal. Surgical tumor enucleation was performed, and the histopathological examination revealed a benign, prepubertal-type teratoma composed entirely of mature elements. Surgical intervention is commonly used for the management of benign testicular tumors in pediatric patients, including prepubertal teratomas. This approach demonstrates an excellent prognosis as it does not elevate the likelihood of recurrence. Prepubertal-type teratomas have rarely been reported in the infantile testis. They may present as a solid mass indistinguishable from the testicle, with no signs of inflammation.

## Introduction

Testicular tumors in pediatrics are rare, with an incidence of 2 cases per 100,000 males. They represent 1-2% of all pediatric solid tumors and present at an average age of 18 months. They are primarily categorized as germ cell tumors (GCTs) (69%) and non-germ cell tumors (31%). Pediatric and adult GCTs have largely similar histological features; however, they differ in the proportion of the subtypes that are represented. In newborns and children, the majority of cases consist of pure yolk sac tumors and teratomas, while seminomas and embryonal carcinomas are uncommon (1-3). Teratomas, the most common tumors found in infants, develop from germ cells and can occur in the sacrococcygeal area, ovaries, testicles, or other sites in the body (4,5). These tumors contain endodermal, mesodermal and ectodermal germ layer derivatives (2). According to the Gonzalez-Crussi histopathological classification, there are three types of teratoma: Mature teratoma (MT), immature teratoma (IT) and malignant teratoma (6). Both mature and immature components are benign, and their potential for metastasis is unclear. Although these lesions exhibit a benign histological appearance, there is a small subset of patients who may face a potentially fatal outcome (6).

As shown by the literature, the majority of cases of MT occur in the intraabdominal region, with only one case of IT observed in the testis (7). The present study reports a rare case of MT in the descended testes of an infant. The case described herein highlights the importance of considering intratesticular MT in the differential diagnosis and demonstrates the role of testis-preserving surgical intervention in managing these cases. The eligibility of the references has been confirmed (8), and the report is organized in line with CaReL criteria and also includes a brief review of the literature (9).

## Case report

### Patient information

A 6-month-old male patient presented Smart Health Tower (Sulaymaniyah, Iraq) with right-sided scrotal swelling, which as noted by his parents. He had no family history of similar medical conditions, and his previous medical and surgical histories were all negative.

### Clinical findings

A physical examination revealed a solid mass without erythema, tenderness, or warmth. The mass was palpable, non-reducible and indistinguishable from the right testicle. It exhibited transillumination and demonstrated no change in size during crying or straining. All the vital signs of the patient were normal, and the patient was pain-free.

### Diagnostic assessment

A scrotal sonography was performed and revealed a large anechoic cystic lesion measuring 30x18 mm within the right scrotal sac. The surrounding testicular tissue appeared stretched and thinned, particularly at its lower pole. No septation, calcification, or solid component was observed. The left testicle appeared to be of normal size and shape. The serum levels of α-fetoprotein (AFP) and β-human chorionic gonadotropin (β-HCG) were within the normal range for his age (AFP, 24.07 IU/ml; β-HCG, <0.100 mIU/ml).

### Therapeutic intervention

**Figure 1:**
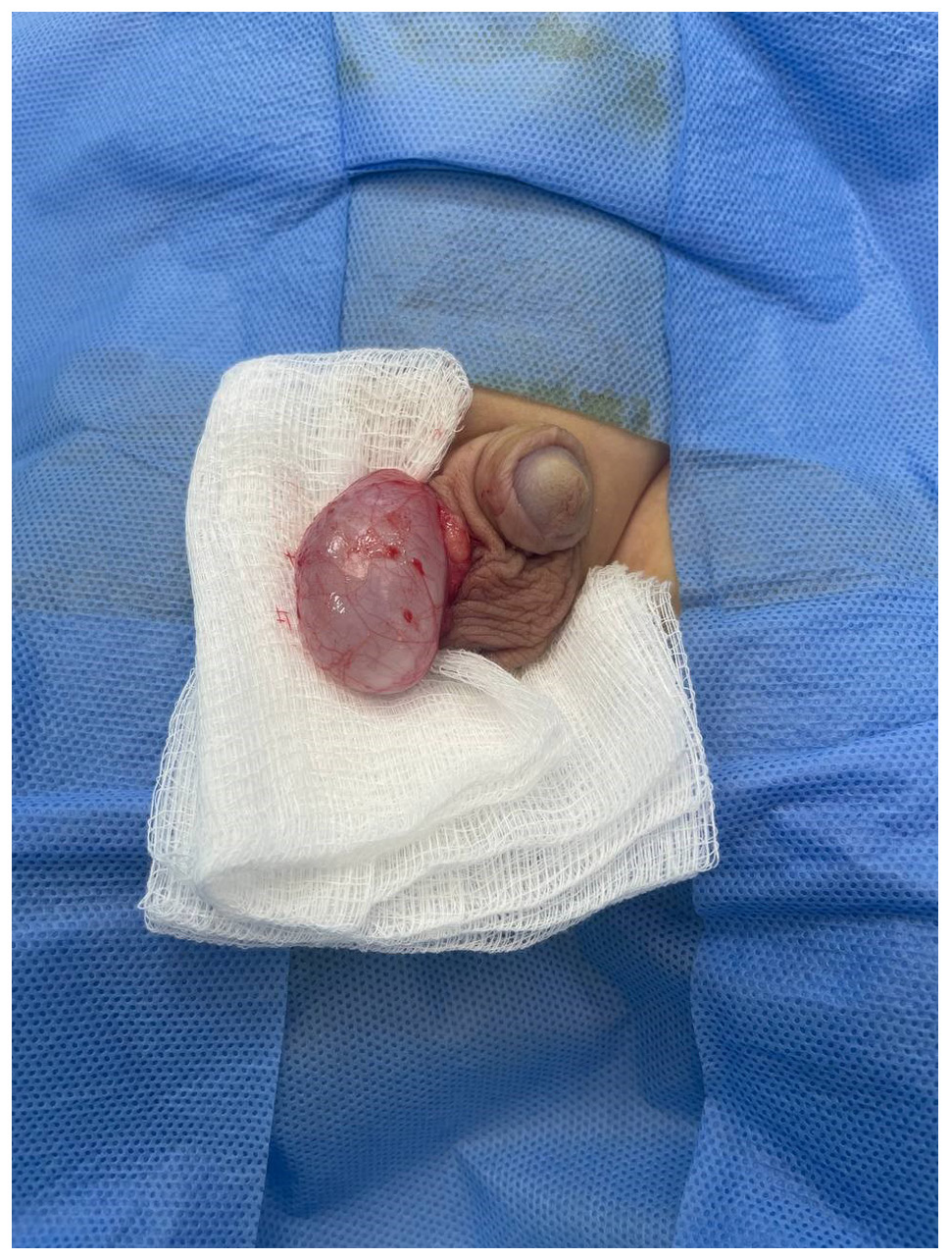
Intraoperatively, there was a 30x18 mm translucent, well-defined, intratesticular cystic lesion.

Testis-sparing tumor enucleation was performed. The tunica albuginea was incised, and the cyst was carefully enucleated intact without rupture (Fig. 1). The remaining testicular parenchyma was sutured, hemostasis was secured, and the testis was returned to its scrotal sac.

**Figure 2:**
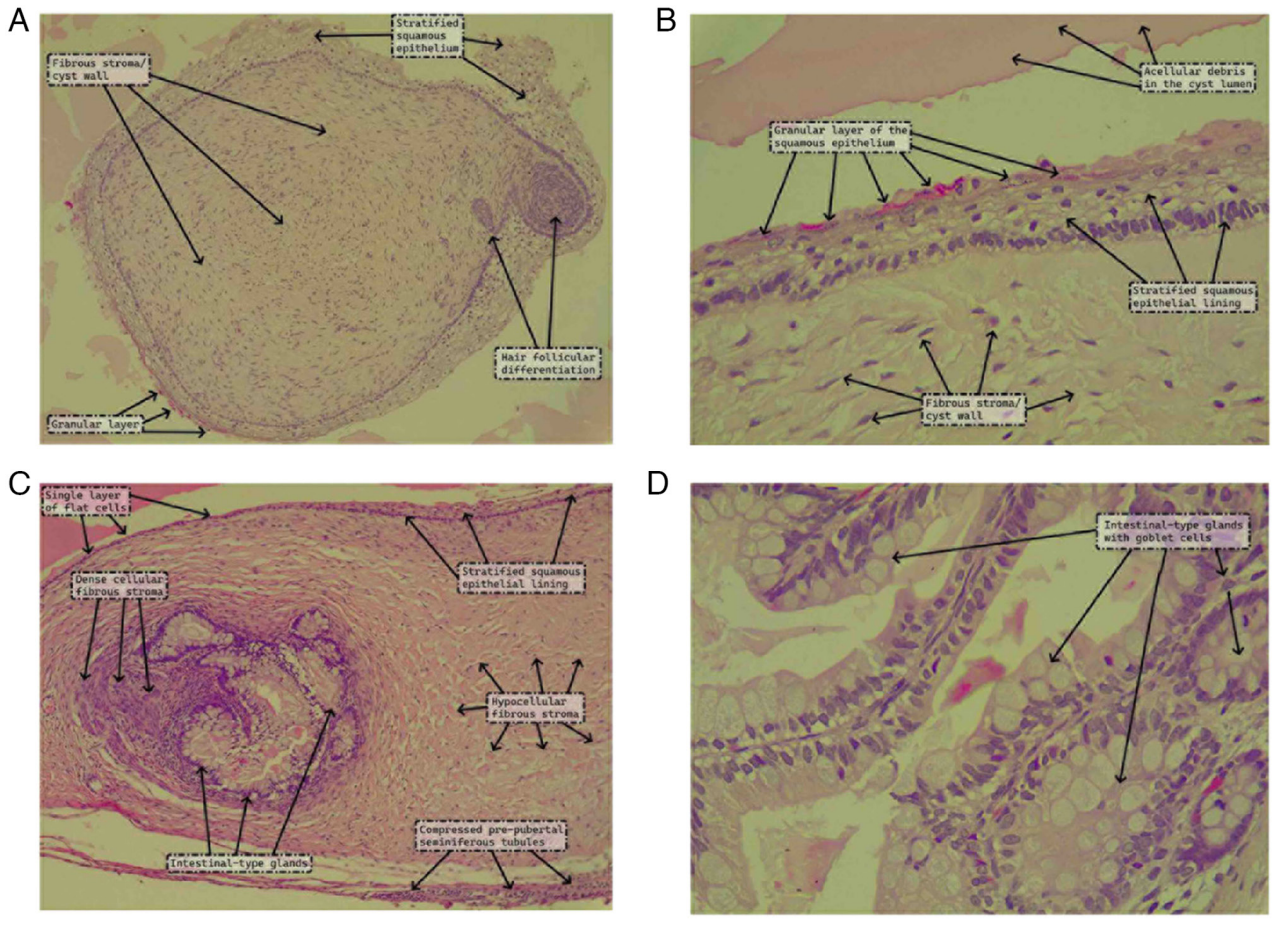
(A and B) The cyst is lined by a stratified squamous epithelial lining that has a patchy granular layer and shows focal hair follicular differentiation, underlaid by a fibro collagenous stroma. The lumen contains acellular debris. (C) The cyst lining exhibits attenuation to a single layer of flat cells adjacent to more stratified squamous areas. The underlying stroma shows focal cellular fibrous condensation. A cluster of intestinal-type glands is present in the wall. (D) The intestinal-type glands are lined by tall columnar cells with lightly eosinophilic cytoplasm and basally located, round nuclei with fine chromatin. There are numerous goblet cells [hematoxylin and eosin staining; (A and C), magnification, x100; (B and D) magnification, x400].

Grossly, the cyst measured 2.7x2.4x2 cm and was unilocular with a shiny, yellow outer surface and a wall thickness of up to 0.3 cm. It was filled with a homogeneous, myxoid/gelatinous material. A microscopic examination was performed. The section was 5-µm-thick and paraffin-embedded. The section was then fixed in 10% neutral buffered formalin at room temperature for 24 h and then stained with hematoxylin and eosin (Bio Optica Co.) for 1-2 min at room temperature and observed under a light microscope (Leica Microsystems GmbH). Upon the microscopic examination, the cyst was found to have an epithelial lining that varied from an attenuated single layer of flat cells to stratified squamous epithelium with a patchy granular layer and focal hair follicular differentiation. The lumen contained acellular debris, while the underlying stroma had a fibro collagenous composition with areas of cellular stromal condensation. There were clusters of intestinal-type glands underneath the cyst lining that were lined by absorptive cells (tall columnar cells with eosinophilic cytoplasm and basal, round nuclei with fine chromatin) and numerous goblet cells (Fig. 2). The prepubertal, immature seminiferous tubules immediately adjacent to the mass were compressed, but there was no germ cell neoplasia in situ, sclerotic tubules, microlithiasis, parenchymal scars, or necrosis. A diagnosis of prepubertal-type MT was thus rendered, and the surgical excision was deemed adequate and sufficient.

### Follow-up

The post-operative period was uneventful; the patient recovered well and was discharged 1 day after the surgery. At 6 to 7 weeks after the surgery, a post-operative ultrasound was performed, which revealed a normal-sized right testis (image not available; data not shown).

## Discussion

Teratoma is a type of embryonic tumor resulting from the improper development of embryonic cells within the primordial germ cells, frequently occurring in primary areas such as the sacrococcygeal and retroperitoneal regions (1,10). In adults, malignant testicular teratoma represents ~60% of all testicular teratomas, while in children, the majority teratomas are of the prepubertal type which are benign (1). These tumors can manifest either as a painful palpable mass or a painless one that is found accidentally, as was the case in our patient (11).

According to the World Health Organization (WHO) classification of tumors, testicular teratomas are classified into the prepubertal-type and postpubertal-type. The prepubertal-type teratomas are usually (although not always) observed in the prepubertal testis, and they are characterized by various forms of somatic tissue derived from any of the three germ layers and often arranged in an organoid pattern and a lack of germ cell neoplasia *in situ*, tubular atrophy (away from the tumor), parenchymal scars, tubular microlithiasis, necrosis and impaired spermatogenesis. They are diploid tumors with a lack of the characteristic chromosome 12p gain observed in post-pubertal-type teratomas and a lack of recurrent somatic mutations. Epidermoid cysts (similar to epidermal inclusion cysts of the skin) and dermoid cysts (similar to their ovarian counterparts) are specialized subtypes of prepubertal-type teratomas that lack other somatic elements. Prepubertal-type teratomas are benign tumors without reports of malignant behavior. Thus, patients are treated sufficiently with surgical excision alone, without the need for systemic adjuvant therapy (12).

The literature review of seven case reports on MT and IT in infants and children revealed 5 cases of MT and only 2 cases of IT, mostly arising from undescended testes within the abdomen. The size of tumors ranged between 2 and 9 cm, with the average age of cases being 15 months (Table I). The present study contributes uniquely to understanding intratesticular prepubertal-type MT in infants compared to cases reported in the literature. Previous studies on prepubertal-type MTs typically involved larger tumors located intra-abdominally, where interventions primarily focused on mass removal, as demonstrated by Yam *et al* (11), Tanaka *et al* (4) and Mukai *et al* (10). Notably, Moritoki *et al* (7) reported only one other case involving an intratesticular location, which required orchidectomy due to the nature of the teratoma. By contrast, the present study documented a rare case of intratesticular MT in infant managed successfully through testis-sparing tumor enucleation.

While yolk sac tumors represent the most common form of GCTs recognized in fully descended testes among prepubertal males, the most common type of teratoma found in intra-abdominal undescended testes is prepubertal-type MT (4,13). In the present study, the prepubertal-type MT appeared in a descended testis. Testicular prepubertal-type MT in the prepubertal age group is typically found in children <5 years of age (11). While the average age of infants with testicular tumors has been reported to be 15 months, the age of presentation in infant cases with intratesticular prepubertal-type MTs is unclear. The age at presentation in the case described herein was 6 months. The accurate diagnosis of intratesticular prepubertal-type MT in infants is crucial due to its rarity and the diverse tissue types involved, and a comprehensive physical examination is essential to differentiate it from hernias and other testicular tissues (11). This accuracy provides the appropriate surgical method and reduces the risk of recurrence. According to reported cases, a precise diagnosis leads to successful tumor enucleation and favorable patient outcomes (1,7,10). An ultrasound examination is essential for evaluating testicular tumors, with evidence indicating that its sensitivity in diagnosing testicular tumors can be as high as 96.6% (1). While an ultrasound plays a vital role, diagnosis can be challenging and may require additional imaging techniques such as computed tomography (CT) or magnetic resonance imaging. A CT scan effectively detects prepubertal-type MT and can provide a provisional diagnosis. The CT imaging features of intra-abdominal testicular prepubertal-type MT are similar to those observed in MTs in other regions of the body (11). A limitation of the present study was the inability to perform a CT scan due to its high cost for the patient's family, and the mass was identified and characterized only through a scrotal ultrasound. Typically, prepubertal-type MT manifests as a well-defined, rounded mass with cystic characteristics (11). The AFP in the serum is released by yolk sac cells during early fetal development, as well as by the proximal small intestine and the liver (1). Its level typically stays elevated, and it only normalizes around the age of 9 months. In the present case, the levels of AFP and β-HCG were normal (5,14).

Surgery has been extensively utilized to treat benign testicular tumors in children, such as prepubertal-type MT, including dermoid cysts. The prognosis of surgical excision of the tumor without orchiectomy is excellent during childhood; it may not increase the possibility of recurrence in cases of benign tumors and is considered safe and feasible (4). Furthermore, since the testis is a vital sexual organ in males, preserving it through surgery holds functional, physiological, and psychological significance for patients with testicular tumors. The only therapeutic intervention performed in the case in the present study for prepubertal-type mature testicular teratomas was testis-sparing tumor enucleation surgery, which was completed without any intraoperative complications (1,15).

The prepubertal-type MT in infants is a benign tumor, and recurrence is uncommon. Radiologic tests are useful for a provisional diagnosis, but they cannot reliably distinguish benign from malignant tumors. Histopathological examination is thus crucial for a definite diagnosis and complete characterization (11).

In conclusion, prepubertal-type MT is a benign tumor that is most commonly found in intra-abdominal testes; however, its occurrence in descended testes, particularly in infants, is rare. It may appear as a solid, non-reducible mass, exhibiting no signs of inflammation or systemic symptoms. The present case report highlights the unusual presentation of an intratesticular prepubertal-type MT in an infant and demonstrates the effectiveness of testis-sparing tumor enucleation. Early diagnosis and surgical intervention are crucial for excellent outcomes with a favorable prognosis and minimal risk of recurrence. Future research is required to emphasize long-term follow-up and the development of standardized guidelines for managing and monitoring benign pediatric testicular tumors.

## Figures and Tables

**Table I:** Review of cases on mature teratoma and immature teratoma in infants and children.

Article no.	Authors, year of publication	Age	Type of teratoma	Study design	Size (cm)	Weight	Location	Intervention	(Refs.)
1	Yam *et al*, 2010	7 Months	Mature teratoma	Case report	7	-	Intra-abdominal	Mass removal	(11)
2	Tanaka *et al*, 2009	2 Months	Mature teratoma	Case report	9	723 g	Intra-abdominal	Mass removal	(4)
3	Pramanik *et al*, 2011	5 Months	Mature teratoma	Case report	4	-	Iliac fossa	Mass removal	(14)
4	Moritoki *et al*, 2019	7 Months	Immature teratoma	Case report	3	-	Testicle	Orchidectomy	(7)
5	Doi *et al*, 2022	5 years	Mature teratoma	Case report	4	30 g	Intra-abdominal	Laparotomy	(13)
6	Hasegawa *et al*, 2006	3 Months	Immature teratoma	Case report	9	380 g	Intra-abdominal	Mass removal	(3)
7	Mukai *et al*, 1998	23 Months	Mature teratoma	Case report	2	-	Intra-abdominal	Mass removal	(10)

## Data Availability

The datasets used and/or analyzed during the current study are available from the corresponding author upon reasonable request.
